# ISCEV standard for clinical visual evoked potentials (2025 update)

**DOI:** 10.1007/s10633-025-10042-1

**Published:** 2025-08-21

**Authors:** M. Šuštar Habjan, M. Bach, M. M. van Genderen, S. Li, A. Mizota, J. Nilsson, D. A. Thompson, A. G. Robson

**Affiliations:** 1 Eye Hospital, University Medical Centre Ljubljana, Ljubljana, Slovenia; 2 Eye Center, Medical Center - University of Freiburg, Faculty of Medicine, University of Freiburg, Freiburg, Germany; 3 Department of Ophthalmology, University Medical Centre Utrecht, Utrecht, The Netherlands; 4 Bartiméus Diagnostic Centre for Complex Visual Disorders, Zeist, The Netherlands; 5 Department of Ophthalmology, First Affiliated Hospital of Xiamen University, School of Medicine, Eye Institute of Xiamen University, Fujian Province, China; 6 Department of Ophthalmology and Optometry, Fujian Medical University, Fujian Province, China; 7 Department of Ophthalmology, Teikyo University School of Medicine, Tokyo, Japan; 8 Department of Clinical Neurophysiology, Sahlgrenska University Hospital, Göteborg, Sweden; 9 The Tony Kriss Visual Electrophysiology Unit, Clinical and Academic Department of Ophthalmology, Sight and Sound Centre, Great Ormond Street Hospital for Children NHS Trust, London, UK; 10 Great Ormond Street Institute for Child Health, University College London, London, UK; 11 Department of Electrophysiology, Moorfields Eye Hospital, London, UK; 12 Institute of Ophthalmology, University College London, London, UK

**Keywords:** Clinical standards, Visual evoked potential (VEP), International Society for Clinical Electrophysiology of Vision (ISCEV), Pattern-reversal visual evoked potential, Pattern onset/offset visual evoked potential, Flash visual evoked potential

## Abstract

Visual evoked potentials (VEPs) are electrophysiologic responses to pattern or flash stimuli, recorded over the occiput. VEPs can provide information regarding the function of the visual system and are valuable in the diagnosis and investigation of optic nerve disease or post-retinal visual pathway dysfunction. The ISCEV VEP Standard specifies stimulus and recording conditions for three basic types of recording: (1) Pattern-reversal VEPs elicited by checkerboard stimuli with large 1° (degree) and small 0.25° check widths. (2) Pattern onset/offset VEPs elicited by checkerboard stimuli with large 1° and small 0.25° check widths. (3) Flash VEPs elicited by a flash which subtends a visual field of at least 20°. The ISCEV VEP Standard protocols are defined for a single recording channel with a midline occipital active electrode. Multi-channel VEPs for evaluation of chiasmal and post-chiasmal lesions, together with protocols specific for pediatric populations, are also described in this document as non-standardized additions. The main changes in the updated ISCEV Standard for clinical VEP include an option to perform a simultaneous pattern electroretinogram (PERG) and pattern-reversal VEP recording, a revised definition of the origin and the analysis of the most prominent VEP components, and more precise descriptions of non-standard multi-channel and pediatric VEP recordings, intended to encourage convergence of widely used non-standard methods. These changes aim to provide a clinically relevant document about current practice which will facilitate good quality recordings and inter-laboratory comparisons.

## Introduction

Visual evoked potentials (VEP) are an established clinical method of assessing the function of the visual pathway from the retina to the primary visual cortex. This document supersedes the ISCEV standard for clinical visual evoked potentials: (2016 update) [1] and specifies stimulus and recording parameters for three basic clinical VEP protocols that can be performed by most clinical electrophysiology laboratories. These are:

1. Pattern-reversal VEP: a response elicited by checkerboard stimuli with large, 1 degree (°) (acceptable range of 0.8° to 1.2°), and small, 0.25° (0.2° to 0.3°) check widths.
2. Pattern onset/offset VEP: a response elicited by checkerboard stimuli with large, 1° (0.8° to 1.2°), and small, 0.25° (0.2° to 0.3°) check widths.
3. Flash VEP: elicited by a flash (brief luminance increment) which subtends at least 20° of visual field.

All ISCEV Standard VEPs are classified as transient VEPs, in which the stimulation rate is sufficiently slow that each response is completed before the onset of a new stimulus. They are defined for a single recording channel with a midline occipital active electrode which is sensitive for the assessment of pre-chiasmal function i.e., function of the eye and/or optic nerves anterior to the optic chiasm. For evaluation of chiasmal and post-chiasmal lesions this document describes a non-standard multi-channel VEP protocol, together with specialized procedures for recording from a pediatric population. ISCEV encourages the use of additional VEP protocols that are not specified in this standard and are valuable to localize dysfunction in the visual pathway or answer specific clinical questions. ISCEV publishes and maintains other Standards for clinical electrophysiological tests, often used to complement VEP testing. These include Standards for full-field ERG testing [2], multifocal electroretinography (mfERG) [3], electro-oculography [4] and pattern electroretinography (PERG) including simultaneous PERG and pattern-reversal VEP [5]. There is also a guideline for calibration and verification of stimuli and recording instruments for use in clinical electrophysiology [6]; a guide to visual electrodiagnostic procedures which highlights the typical clinical applications of all ISCEV standard tests including the VEP [7]. In addition, extended protocols are published and may be indicated for enhanced or supplementary characterization, including an extended protocol for VEP methods of estimation of visual acuity [8]. Other extended protocols include the dark-adapted red flash ERG [9], photopic On–Off ERG [10], S-cone ERG [11], the stimulus response series for light adapted full-field ERG [12] and dark-adapted full field ERG b-wave [13], the photopic negative response (PhNR) of the full-field ERG [14] and an extended protocol for derivation and analysis of the strong flash rod-isolated ERG a-wave [15]. The ISCEV website should be consulted for updates (iscev.org/standards). This document is not a safety standard, and it does not mandate procedures for individual patients nor define the qualifications required of those administering or interpreting the tests.

### Summary of changes to the ISCEV VEP standard

This document updates the 2016 version of the ISCEV Standard for clinical VEP testing [1]. The major change in this document is the inclusion of the option to perform an ISCEV Standard simultaneous PERG and pattern-reversal VEP recording. In addition, this Standard provides a description of the origin of individual VEP waveform components, their analysis and typical clinical applications. In the description of pattern-reversal VEP components, the N135 component was renamed to N145, which is more consistent with the literature. The stimulation rate for flash VEP was changed to 1–2 Hz, allowing for faster recording. Non-standard methods of multi-channel and pediatric VEP are described in more detail and the document is also formatted to be more consistent with other ISCEV Standards, by including sections on the stimulus field size, data display system, visual display units (VDUs) for stimulation and refraction. These changes aim to provide a clinically useful document which will facilitate good quality recordings and inter-laboratory comparison.

### Overview and origins of VEP

The generation of the VEP signal depends on a complex sequence of optical and neural events. The stimulus passes through the ocular media to activate the photoreceptors and their connecting neurons. Retinal ganglion axons transmit this signal along the optic nerve of each eye. Approximately 50% of the axons of each eye then decussate at the chiasm to project to the contralateral hemisphere of the brain; the remaining 50% project to the ipsilateral hemisphere. These optic nerve axons make their first synapse after the chiasm at the lateral geniculate nucleus of the thalamus from which the visual signal is transmitted along the optic radiations to reach layer 4 of area V1 of the primary visual cortex (striate cortex). The central visual field is retinotopically mapped to areas of the occipital pole, whereas the peripheral visual field is represented deeper within the calcarine sulcus. Consequently, the VEP signal is dominated by macular field projections.

The pattern-reversal VEP is elicited by an alternating checkerboard pattern composed of black and white checks, with constant mean luminance. The response waveform has an initial negative polarity N75 component that occurs at around 75 ms after the reversal, followed by a positive component P100 at approximately 100 ms, and a negative N145 at around 145 ms. The P100 is usually the most prominent and robust component with the highest clinical importance. It is thought to originate mostly from neuronal generators in the striate cortex, although some studies attribute contributions from extra-striate visual areas. The N75 also has its origins in the striate cortex. Most of the pattern-reversal VEP signal arises from macular fibers of the optic nerve and covers up to 10° of the central stimulus field. The reversal VEP waveform is influenced by check width, with relatively small checks (e.g., 0.25°) enabling a more selective evaluation of the foveolar projection than the response to large checks (e.g., 1°). It is noted that small checks are also more sensitive to factors that degrade the optical quality of the stimulus, such as non-optimal refraction.

The pattern onset/offset VEP is elicited by the appearance/disappearance of a checkerboard pattern with constant mean luminance i.e., there is alternation between a zero and higher level of contrast. At the stimulus onset, waveform usually consists of three main peaks: a positive peak between 60 and 110 ms after pattern onset (C1), followed by a negative peak at 80–150 ms (C2) and a positive peak at 100–250 ms (C3). Additionally, a negative–positive–negative complex appears at the offset of the stimulus, these peaks can be labelled as C4, C5 and C6. There may be high variability in the response waveform, which is age-dependent, the appearance and peak time of individual components may also depend on the check width (see Sect. "VEP waveshape in childhood"). The origin of pattern onset/offset VEP has yet to be fully established, but early components are most likely generated in the striate cortex.

The flash VEP is elicited by a luminance flash and is typically characterized by negative and positive polarity components labelled in order of appearance, with N2 and P2 usually most prominent in adults. The peak times of N2 component typically occur between 60 and 120 ms and for P2 between N2 and 150 ms.

It is emphasized that the peak time ranges for pattern reversal, pattern onset/offset and flash VEPs described above are approximate, and do not define reference limits for clinical use.

## Clinical applications of the standard VEP

This Standard describes three protocols for the objective assessment of optic nerve and post-retinal visual pathway function. The choice of protocol/s should be informed by the reasons for testing and clinical circumstances.

The pattern-reversal VEP is usually the method of choice for the assessment of optic nerve function. The pattern-reversal VEP shows least inter-subject variability in waveform and timing compared to VEPs elicited by other stimuli. Pattern-reversal VEP abnormalities are not specific and can be caused by macular dysfunction, poor compliance, media opacity, sub-optimal refraction and nystagmus, in addition to optic nerve and cortical dysfunction. Reliable interpretation of an abnormal pattern-reversal VEP often requires functional assessment of retinal macular function with PERG or mfERG.

The pattern onset/offset VEP has specific applications e.g., for the assessment of patients with nystagmus. Pattern onset/offset VEPs are also useful in the detection of non-organic visual loss or in cases of malingering, as accommodative defocus of pattern onset/offset stimuli is typically more difficult than for pattern reversal stimuli, especially if the onset duration is short.

The flash VEP is generally less sensitive to optic nerve dysfunction than the pattern VEP. It is useful when there is significant media opacity or poor optical quality, limited cooperation or an inability to fixate. Flash VEPs can also be used to assess visual pathway function when pattern VEPs are non-recordable. There is wider inter-subject variability of flash VEP waveforms than for pattern VEPs. Flash VEPs may reveal additional clinically relevant information and complementary use of both flash and pattern stimulation may be appropriate in some patients.

Multi-channel VEPs, as an addition to, or incorporating, the ISCEV Standard midline VEPs, are needed to detect dysfunction of the optic chiasm and/or retro chiasmal visual pathways. The clinical applications of VEPs and other complementary electrophysiological techniques are further described in the ISCEV guide to visual electrodiagnostic procedures [7].

## Technology

Standard equipment for visual stimulus generation, amplification of physiological signals, and the recording and storing of electrophysiologic data is required for VEP recording. Information about the calibration of equipment and measurement of the stimulus and recording parameters is included in the ISCEV guideline for calibration and verification of stimuli and recording instruments [6].

### Electrodes

#### Recording electrodes

Skin electrodes such as silver–silver chloride or gold cup electrodes are recommended for recording VEPs. It is recommended that the active occipital electrode is connected to the positive input of the recording system and the reference electrode to the negative input.

#### Electrode placement

**Fig. 1:**
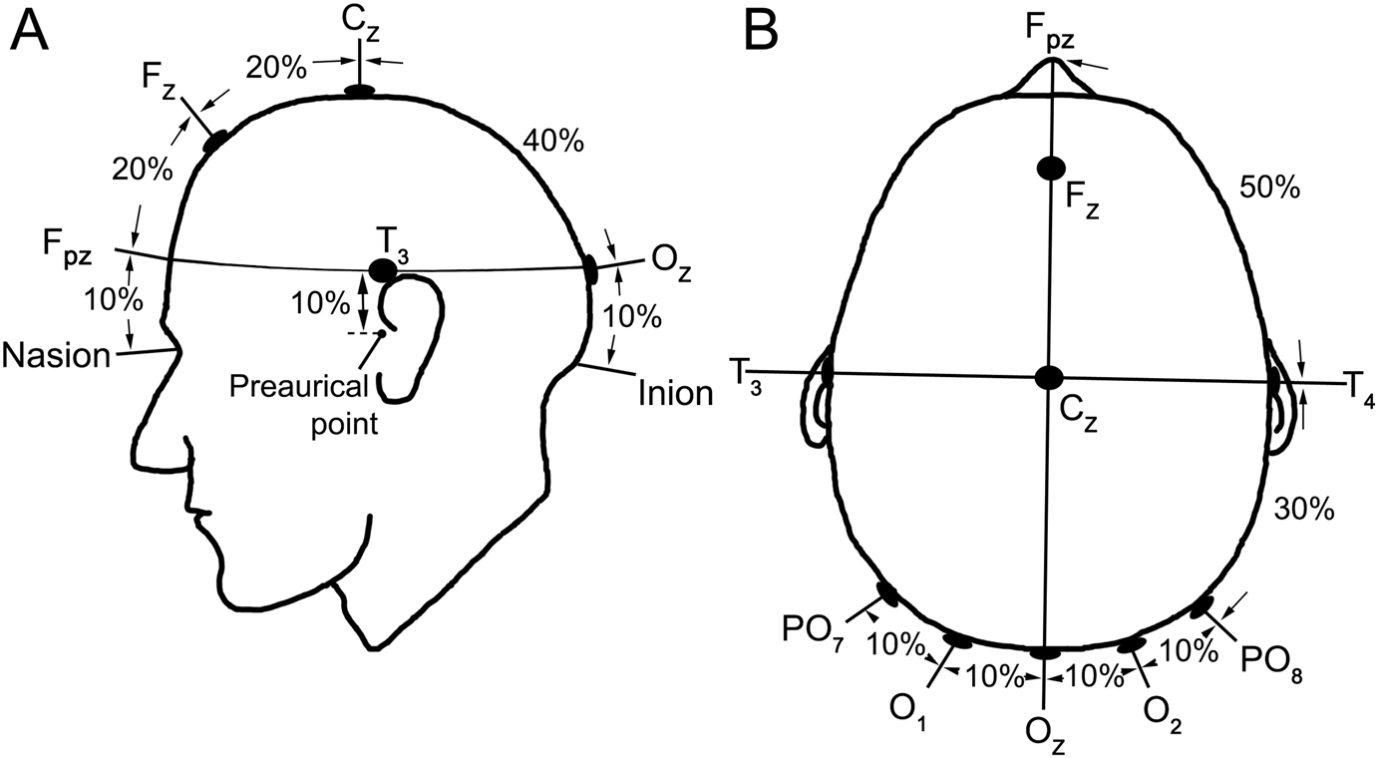
Electrode locations. **A** Location of active and reference electrodes for Standard VEPs. The active electrode is located along the midline at Oz (10% of the nasion-inion distance above the inion). The reference electrode is located at location F_z_ (30% of the nasion-inion distance above the nasion). Ground electrode positioning is not critical, but a typical location is on the vertex (position C_z_), forehead, earlobe or mastoid. The subscript z indicates a midline position. **B** Locations of the lateral active electrodes for multi-channel VEP recording. Positions T_3_ and T_4_ are placed at the intersection of two measurements; 10% of the distance between right and left preauricular points intersecting the vertex. For multichannel recordings electrodes are positioned on locations PO_7_, O_1_, Oz, O_2_ and PO_8_, separated by 10% of the distance between O_z_ and F_pz_, according to an arc intersecting T_4_ or T_3_

The scalp electrodes should be placed relative to bony landmarks, in proportion to the size of the head, based on the International 10–10 system [16] (see Fig. 1). The anterior/posterior midline measurements for electrode positions are based on the distance between the nasion and the inion over the vertex, with the vertex midway between the right and left pre-auricular points. The active electrode is placed on the occipital scalp over the visual cortex at position O_z_ with the reference electrode at the mid-frontal position F_z_. A separate single electrode should be attached and connected to the ground. Typical positions for the ground electrode include the vertex (C_z_), forehead, earlobe or mastoid.

#### Electrode characteristics

The skin should be prepared by cleaning, and a suitable paste or gel used to ensure good, stable electrical connection. The impedance of passive skin electrodes measured between 10 and 100 Hz should normally be 5 kΩ or less. For maximal suppression of mains interference, the recording and reference electrodes should have similar impedance levels, which should differ by no more than 1 kΩ between electrodes. Especially in young children, a headband over the electrodes may be helpful to allow stable electrode connections throughout the examination.

#### Electrode stability

In the absence of stimulation and eye movement, the baseline voltage should be stable. It is recommended that the raw input trace is monitored. In order to achieve stability, the electrodes may need to be non-polarizable e.g., silver-silver chloride electrodes.

### Stimuli

Standard stimulus and recording conditions for each of the three Standard VEP protocols are described below and are summarized in Table 1.

**Table 1:** ISCEV VEP Standard stimulus parameters. Values ​​in parentheses indicate tolerances

(a) Standard stimuli							
Stimulus type	Field size	Presentation	Stimulus	Mean luminance (cd·m^−2^)	Michaelson contrast (%)	Presentation rate	Acquisition time
Pattern-reversal	15° (± 3°)	Monocular	Check widths: 1° (0.8°–1.2°); 0.25° (0.2°–0.3°)	50 (40–60)	≥ 80	2 reversals/s (1.8–2.2 reversals/s)	250 ms
Pattern onset/offset	15° (± 3°)	Monocular	Check widths: 1° (0.8°–1.2°); 0.25° (0.2°–0.3°)	50 (40–60)	≥ 80	200 ms on; 400-500 ms off	500 ms
Flash stimulation	≥ 20°	Monocular	Flash strength: 3 cd·s·m^−2^ (2.7–3.3 cd·s·m^-2^)	-	-	1–2 Hz (0.9–2.2 Hz)	250 ms
Optional simultaneous PERG and VEP	15°(± 3°)	Monocular	Check width: 0.8°–1.0°	50 (40–60)	≥ 80	3 reversals/s	250–285 ms

(b) Standard recording						
	Electrode montage (international 10–10 channel system)	Filters (-3 dB)				
		Active	Reference	Low freq	High freq	Sweeps averaged
Pattern stimulation		Oz	Fz	≤ 1	≥ 100	≥ 50
Flash stimulation		Oz	Fz	≤ 1	≥ 100	≥ 50

#### Pattern stimulation

All Standard pattern stimuli are high-contrast, black-and-white checkerboards consisting of squares with equal sides. The viewing distance is typically between 50 and 150 cm, adjusted to obtain the required field size for any physical size of display screen. Stimulus changes, whether pattern reversal or onset/offset, must be achieved without a change in the mean luminance of the stimulus. Both transient and step changes in luminance (luminance artifacts) will influence VEPs by luminance intrusion. Such luminance artifacts in stimuli may be detected by viewing the stimulus through a sheet of white paper, to check for a visible flash or flicker [6].

#### Visual display units (VDUs) for stimulation

The technology of VDUs used to present pattern stimuli may affect stimulus definition and timing and not all are suitable for use as pattern stimulators for VEP recording. Major types of VDU and other optical imaging systems used in clinical visual electrophysiology are compared in the ISCEV guidelines for calibration and verification of stimuli and recording instruments [6], including cathode ray tube (CRT), liquid crystal display (LCD), organic light emitting diode (OLED) and other technologies. The frame rate of the VDU is a significant stimulus parameter for VEP recording. For CRTs, a frequency of 75 Hz or greater should be used. Most current LCDs present a static, non-flickering image, typically refreshed at 60 Hz, but there are luminance artifacts during pattern modulation. As such, they are unsuitable for pattern VEP recording unless special precautions are taken (such as added compensation corrections and verified or calibrated stimuli). Rarely used but possible are projection systems, plasma displays and displays using OLEDs to create the image. LCD-based displays and projectors may have a significant delay between signal input and stimulus output. This delay must be considered when defining ‘time zero’ of the VEP (usually available from the manufacturer or can be measured with a photodiode).

The frame rate or refresh rate affects VEP peak time, because time zero is historically defined as the time at which the refresh begins at the top of the screen. To standardize responses across different VDUs/different laboratories, it is better to define time zero as the time when the center of the screen is updated. When switching to this definition, VEP peak times are shorter by a well-defined interval (e.g., 6 ms) and independent of refresh rate. It is essential to be aware of the refresh rate and the screen location that is defined as time zero, to allow peak times to be interpreted correctly and compared between laboratories.

#### Field size and check width

Patterned stimuli are defined by the visual angle subtended by the side of a single check in degrees (°) or minutes of arc (min) subtended at the eye (1° = 60 min of arc). Two check widths are specified: 1° (with an acceptable range of 0.8° to 1.2°) and 0.25° (0.2° to 0.3°). All checks should be square, and there should be an equal number of light and dark checks. It is not necessary to use a square stimulus field, but the aspect ratio between the width and the height of the stimulus field should be between 4:3 and 1:1. The mean of the width and the height of the stimulus field should be 15° (± 3°). The black and white checks should be located so that the corners of 4 checks (node) are positioned at the center of the field. A fixation mark is placed at this point. The size of fixation mark should be limited, to avoid too great a loss of the central macular contribution to the response.

#### Screen luminance

A mean photopic luminance of 50 cd·m^−2^ (with an acceptable range of 40–60 cd·m^−2^) is required, with the photopic luminance for the white areas of 80 cd·m^−2^ or more. The mean luminance of the stimulus screen must be constant during checkerboard reversals and onset-offset phases, i.e., without a transient luminance change. The luminance of the stimulus should be uniform between the center and the periphery of the field. However, many optical and electronic systems do not provide truly uniform fields; luminance variation from center to periphery should be below 30%.

#### Contrast

Contrast between black and white squares should be maximal (close to 100%) and not less than 80%, as defined by Michelson contrast.

$${{Michelson }}\;{{contrast }} = 100\%\cdot\frac{L_{max}-L_{min}}{L_{max}+L_{min}} $$

where Lmax = maximal luminance (bright squares), Lmin = minimal luminance (dark squares).

The contrast of the stimulus should be uniform between the center and the periphery of the field and measured in the conditions of ambient lighting used for testing.

#### Pattern-reversal stimuli

For the pattern-reversal VEP, the black and white checks change phase (reverse) abruptly (i.e., black to white and white to black) with no overall change in the luminance of the screen. To meet this requirement, there must be equal numbers of light and dark checks in the display. This will help ensure there are no local luminance changes with each reversal. Standard pattern-reversal VEPs should be obtained using a reversal rate of 2.0 ± 0.2 reversals per second (rps) (this corresponds to 1.0 ± 0.1 Hz, as a full cycle includes two reversals). Reversal rate must be reported in rps, not in Hz. For simultaneous recording of PERG and pattern-reversal VEP see Sect. "Simultaneous PERG and pattern-reversal VEP".

#### Pattern onset/offset stimuli

For pattern onset/offset VEP, the checkerboard pattern is abruptly exchanged with a diffuse gray background. The mean luminance of the diffuse background and the checkerboard must be identical with no change of luminance during the transition. Pattern onset duration is 200 ms separated by 400-500 ms of diffuse background. This ensures separation of onset and offset VEP components. The data acquisition system must be triggered by stimulus onset and indicate the onset of the stimulus.

#### Flash stimulus

The flash VEP is elicited by a brief flash (≤ 5 ms), presented at a flash rate of 1–2 per second (1.0–2.0 Hz; acceptable range 0.9–2.2 Hz). The flash may be delivered by a ganzfeld stimulator. The strength (time-integrated luminance) of a ganzfeld flash stimulus should be 3 photopic candelas seconds per meter squared (cd·s·m^−2^) with an acceptable range of 2.7–3.3 cd·s·m^−2^, as incorporated within the ISCEV Standard full-field ERG protocol [2]. Non-ganzfeld or handheld flash stimulators subtending an angle of at least 20 degrees may also be used to evoke standard flash VEPs, but reference data must be obtained using identical stimuli, with a stimulus strength and methods that ensure similar waveforms to those evoked by the ganzfeld flash. The method of delivering flash stimuli and stimulus parameters must be stated. For some applications, e.g., for assessing integrity of the visual pathway in the presence of a dense media opacity, additional stronger or dimmer flashes may be indicated but this should be stated in the report.

#### Background ambient illumination

The background level of luminance or ambient light is not critical when using standard VEP techniques, e.g., ambient dim room lighting may be used, but it is essential that it closely replicates the conditions used for the collection of reference data. Care should be taken to keep bright lights out of the subject's direct view and away from the VDU, where it may produce veiling glare and reduce the stimulus contrast.

### Recording equipment

#### Patient isolation

Electrical isolation of the patient should be ensured according to the current safety standards of the country in which the test is performed. In the absence of national requirements, the equipment should meet a general standard, such as Medical Electrical Equipment IEC 60601-1.

#### Amplification

Amplifiers with a minimum input impedance of 10 MΩ are required. The system should be capable of recording frequencies across a broad range, and a band-pass filter should be applied that includes the minimum frequency range of 1–100 Hz (corner frequencies defining 3 dB attenuation). Users should be aware that changes to the filter characteristics or corner frequencies of the high-pass and low-pass filters may change the amplitude and peak times of the VEP. Notch filters (that suppress signals at the main line frequency) are contraindicated, as they may reduce or distort the signal. Some users may encounter severe electromagnetic interference from the stimulus display that makes it difficult to obtain satisfactory recordings with these filter settings. Ideally, such interference should be eliminated e.g., by shielding, by ensuring the impedance of the electrodes is sufficiently low and similar, and by bundling or twisting together the electrode leads to minimize the influence of electromagnetic fields.

#### Averaging and signal analysis

Signal averaging is necessary because of the small amplitude of the VEP compared to electroencephalographic (EEG) activity and other sources of noise. The number of stimulus presentations (sweeps) required per average depends upon the signal-to-noise ratio. In most clinical settings, the minimum number of sweeps per average should be at least 50. Up to 100 sweeps or more may be needed when the VEP is small or undetectable, or in conditions with high levels of background noise. Interrupted averaging may prevent fatigue in these circumstances. A minimum of two averages must be recorded and displayed to demonstrate reproducibility i.e., at least one close replication. Several recordings may be needed to establish consistency and to enable meaningful interpretation. If post-hoc filtering is used to present cleaner waveforms, then the filtering process (i.e., bandwidth, filter type) should be stated. If response reproducibility is poor, adding a trial without a stimulus can help distinguish between a true response and an apparent response due to high noise.

In addition to monitoring the raw signal, it may also be useful to monitor the ongoing average signal or to utilize interleaved averaging (also known as odd–even averaging). The latter technique generates two sub-averages, one from the even sweeps, the other from the odd ones. Both sub-averages can be displayed simultaneously and overlaid on the screen, allowing for a more reliable assessment of the repeatability of the response in real time. In such a way, fatigue and other factors do not affect one sub-average more than the other.

#### Artefact rejection

Computerized artefact rejection is essential. The limits for rejection should be set at a maximum of ± 100 μV, although increasing the limit is acceptable in subjects with extremely high amplitude VEPs. Any post hoc manual rejection of individual sweeps confounded by obvious artefacts such as eye movement and blink, which may deflect the pre-baseline recording or cause the trace to fall out of the response window, should use a consistent criterion of exclusion that leaves sufficient sweeps for an average.

#### Sampling rate

A minimum sampling rate of 1000 Hz (1 ms per sample) is recommended. See the ISCEV guideline for calibration and verification of stimuli and recording instruments [6] for further information.

#### Analysis time

The minimum analysis time (sweep time) for all adult transient flash and pattern-reversal VEPs is 250 ms post stimulus. For onset/offset VEPs, the recommended analysis time is 500 ms to examine offset components. The VEP in infants has longer peak times; a sweep duration of up to 400 ms is often necessary to adequately visualize typical pattern-reversal or flash VEP waveforms in those under 6 months of age (see Sect. "Pediatric VEP recording"). A pre-stimulus baseline of 20 ms or more may be used to facilitate detection and compensation of any baseline drift.

#### Data display system

Display systems must have adequate resolution to accurately represent the characteristics of the VEP signal. Ideally, the recording system should provide simultaneous display of the input signal and the accumulating average. In the absence of a simultaneous display, frequent alternation between the input signal and ongoing average is advisable, so that the quality of the recordings can be adequately monitored. Even with a computerized artefact rejection system, it is important that the input signal is continuously monitored for baseline stability and absence of amplifier saturation.

#### Calibration

All stimulus parameters including luminance and contrast should be calibrated either locally or by the manufacturer. Calibration of amplifier gain is assessed by passing a known signal, with amplitude and timing in the range of the physiological signals through the entire system. Calibration methods and recommended frequency of verification for different types of display are specified in the ISCEV guidelines [6].

## Clinical protocol

### Preparation of the patient

#### Positioning, pupils and preadaptation

Patients should be as comfortable as possible with their head in a stable position. A headrest may be helpful but contact with the electrodes should be avoided, as this may cause artefacts. The pattern-reversal and pattern onset/offset VEP protocols should be recorded without dilation of the pupils to maximize retinal image quality. In case of extreme pupil size (miosis, mydriasis) or anisocoria, pupil diameters should be documented. A preadaptation period is not necessary, but procedures that may reduce optical quality should be avoided prior to VEP testing.

#### Fixation

For pattern stimulation, the patient should be instructed to maintain a stable central fixation on the fixation mark. If unable to see the fixation mark, patients should be directed to look towards the center of the screen and to keep their eyes still. Cross hairs may be more suitable in cases of central scotoma. Fixation should be closely monitored, either with a camera that allows visualization of the pupil or directly by the observer/practitioner. Stable fixation is less important for flash VEPs. Excessive blinking during recording should be discouraged and pausing of the recording (interrupted averaging) may be advantageous for some patients. In case of dry eyes, additional application of methylcellulose based artificial tears or buffered saline drops may improve patient comfort and compliance.

#### Refraction

Optimal refraction is necessary for pattern-reversal and pattern onset/offset VEP, but not for flash VEP recording. Patients can wear habitual correction for the test distance, trial lenses may also be used. Bifocals or progressive glasses may not ensure optimal imaging over the full stimulus field. Tinted and photochromatic glasses change peak time because they reduce luminance and should be avoided. The spectacle lenses need to be clean to prevent contrast loss due to stray light. Optimal correction is particularly important for short viewing distances in patients with reduced accommodation, such as patients with presbyopia (optimal focus at 100 cm viewing distance requires an additional + 1 diopter and at 50 cm + 2 diopters). The use of refractive correction should be noted.

#### Monocular and binocular recording

Monocular stimulation is standard for all VEP protocols. During monocular stimulation care should be taken to ensure that the stimulus is unilateral. A light-tight opaque patch over the untested eye is usually essential for flash VEP. In some infants or other special populations where monocular stimulation cannot be performed, binocular stimulation may have value in assessing visual pathway function, but use of this non-standard method should be clearly acknowledged in reports.

### The analysis of the ISCEV standard VEP waveforms

**Fig. 2:**
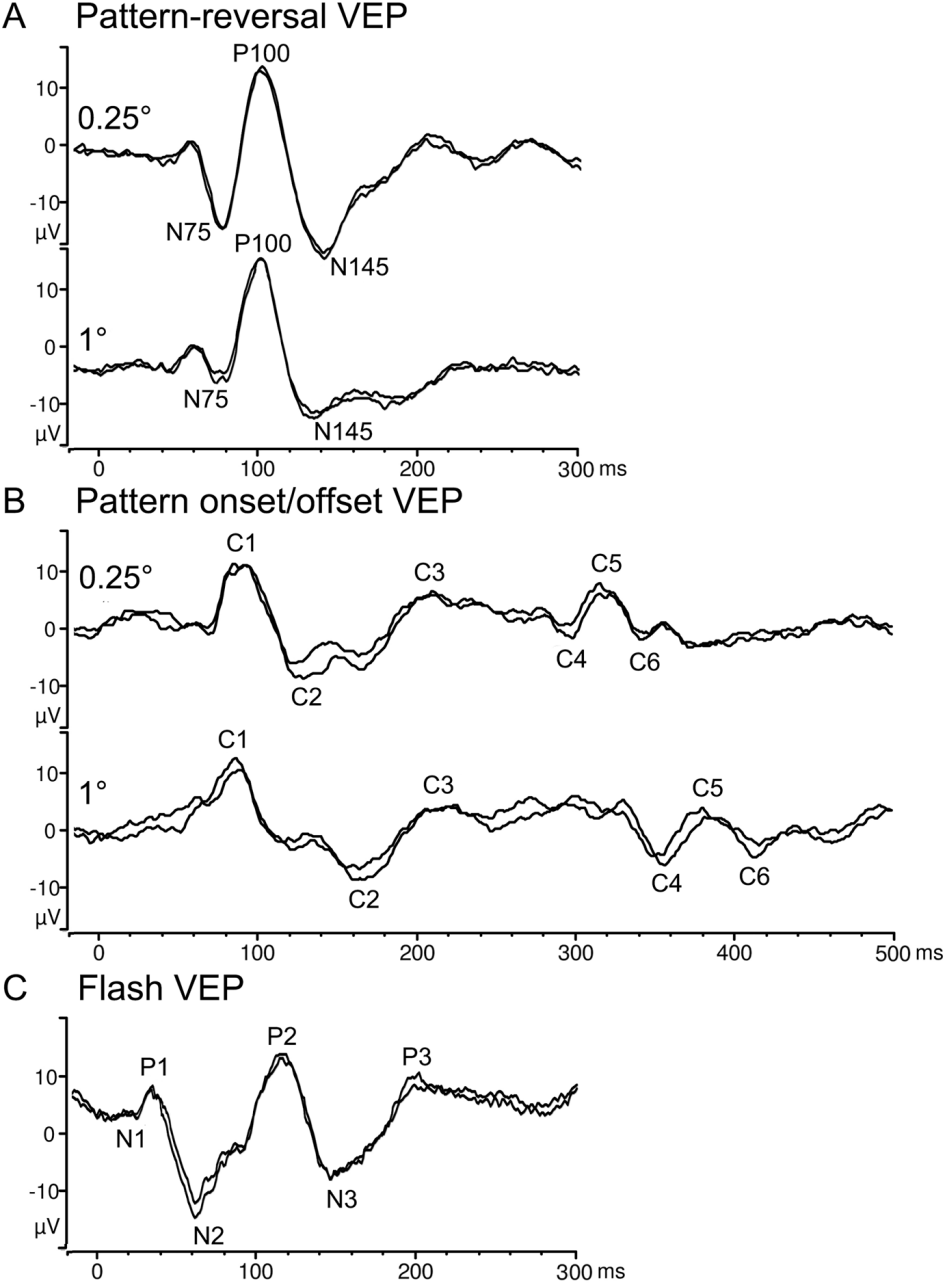
Pattern-reversal (**A**) and pattern onset/offset VEPs (**B**), each elicited with 0.25° and 1° checks, and a flash VEP (**C**), all recorded from the same adult subject, with superimposed waveforms to demonstrate reproducibility. The most prominent components of each response are indicated. The waveforms are examples and do not indicate minimum, maximum or typical values. Note that pattern onset/offset VEP requires a 500-ms sweep to visualize both onset and offset components

VEP waveforms are influenced by age and are illustrated below for an adult subject (Fig. 2). The amplitudes of all standard VEP components are measured between the peaks and troughs of a grand average of the most consistent waveforms. The time from the stimulus onset or reversal to the peaks or troughs is described as the peak time. Historically, the term latency has been used in VEP studies to indicate the time from stimulus onset to the peak of a component, but this is discouraged, since the term ʺlatencyʺ also means the time from the onset of the stimulus to the beginning of a response.

#### Pattern-reversal VEP

A typical pattern-reversal VEP waveform consists of negative and positive peaks named N75, P100 and N145, based on typical mean peak times (see Fig. 2A). P100 is usually the most prominent positive peak and shows the least variation between subjects, a high degree of interocular symmetry and a relatively high degree of inter-session stability. The standard measure of pattern-reversal VEP amplitude is the difference between P100 from the trough of the preceding N75. P100 peak time should be measured from the start of the contrast reversal.

#### Pattern onset/offset VEP

The VEP elicited by standard pattern onset/offset stimulation typically consists of a positive peak after the stimulus onset, followed by a negative and another positive peak (Fig. 2B). The nomenclature of these three peaks is customarily termed C1, C2 and C3, respectively. Additional peaks also appear at the offset of the stimulus, this negative–positive–negative complex can be defined as C4, C5 and C6. If there is uncertainty in identifying specific components, other naming conventions are also acceptable but should be clearly defined by the user. There may be high variability in the response waveform, so such peak descriptions may not apply. Analysis of the most prominent component is acceptable but must be specified and compared with appropriate reference data. Amplitudes are measured from the preceding positive or negative peak.

#### Flash VEP

The typical VEP to standard flash stimulation consists of a series of negative and positive waves. Major peaks are designated as negative and positive in a numerical sequence (see Fig. 2C). The most consistent and robust components of the flash VEP in adults are the N2 and P2 components. Measurements of P2 amplitude should be taken from the positive P2 peak to the preceding N2 negativity. Flash VEPs show greater inter-subject variability than pattern VEPs but usually show a high degree of inter-ocular symmetry.

### VEP reporting

#### VEP interpretation

**Fig. 3:**
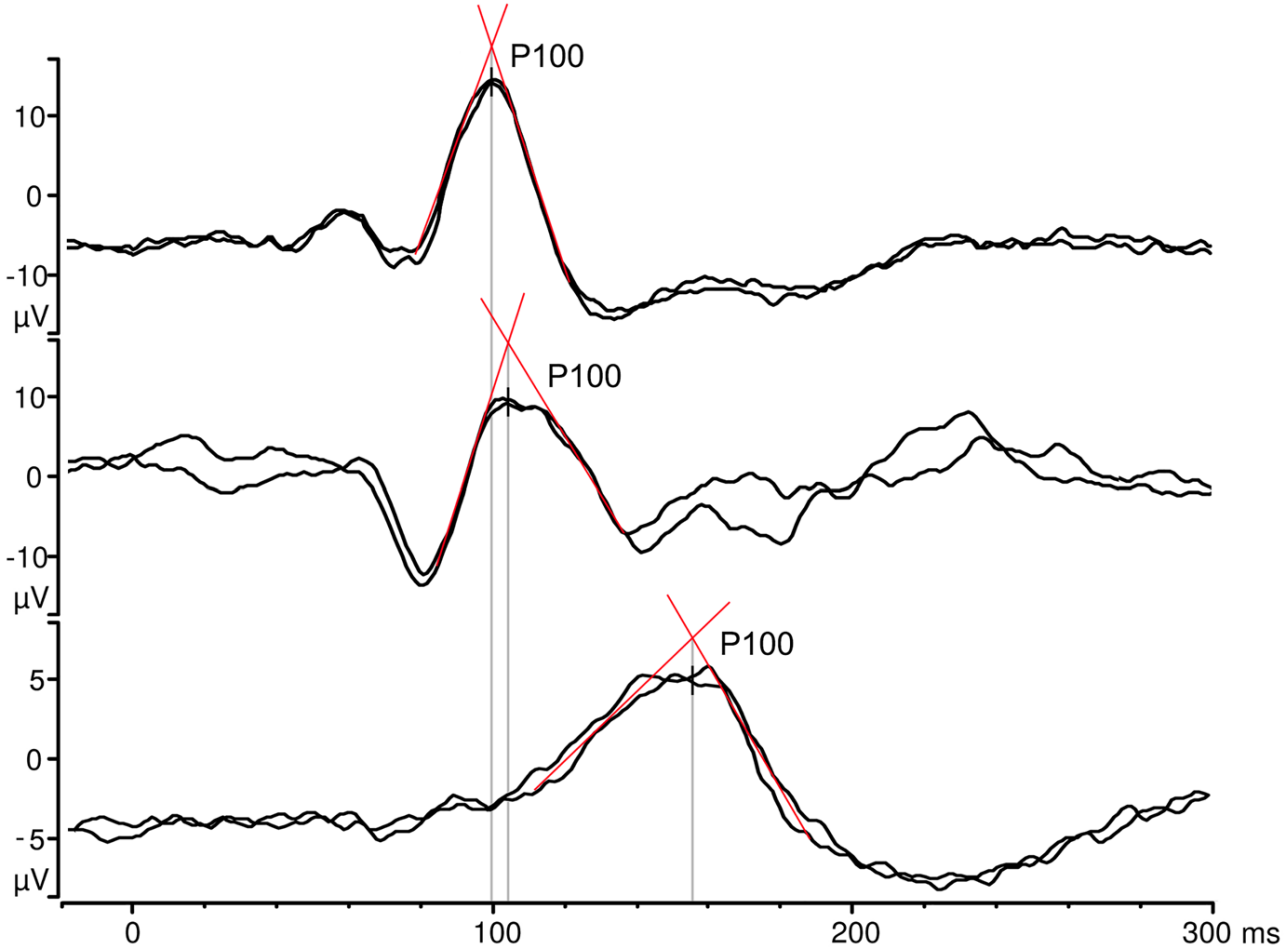
The triangulation method—the peak time is determined at the intersection of the tangents, drawn according to the slope of the ascending and descending limbs of the response

VEP abnormalities are not specific and can occur in a wide variety of ophthalmological and neurological conditions. The interpretation should be informed by comparison with reference data or between the eyes, or with previous recordings. The analysis of the peak time, especially in pattern-reversal VEP, requires special attention. Many peaks are not symmetric or regular, and the highest point of the peak does not necessarily represent the mid-point of the response, especially if additional noise is present in the recording. In such cases it may be valuable to use a triangulation method, where tangents are drawn to the slope of the upward and downward sides of the peak and the peak time is estimated at the intersection of these tangents (Fig. 3).

#### VEP reporting

It is recommended that all reports contain measurements of amplitude and peak time of the most prominent VEP components where possible, e.g., P100, C1 and P2 along with their 95% reference intervals. The report should also include a description of any waveform abnormality, such as a bifid or distorted shape. In the instance of a bifid pattern-reversal VEP waveform with a complex positive–negative-positive (P-N-P) configuration (usually associated with reduced macular field sensitivity) it is helpful to describe polarity and peak time of the configuration, for example P75-N105-P135, adding the amplitude of the most prominent positive peak rather than labelling one of the positive peaks as P100. The reports should specify the pattern check width (for both large and small checks), stimulus rate (in reversals per second), number of sweeps averaged, mean luminance, Michelson contrast, field size and the eye tested. Traces should have a clear indication of polarity and indicate time in milliseconds and amplitude in microvolts. Although not essential, it is recommended that VEP traces are displayed with the positive polarity upwards (in agreement with all other electrophysiological responses covered by the ISCEV standards). Confounding factors such as poor fixation, eye movements, or suspected defocus should be noted. Reports should indicate compliance with or specify departures from this Standard. VEP reports involving non-standard protocols must be clearly acknowledged and described e.g., including details of the stimuli, electrode positions or the combination of the electrode positions used. The abnormality in the VEP should be described and considered in light of other visual electrodiagnostic results.

#### Reference ranges

Establishing laboratory-specific VEP reference values is the optimal process, which involves recruiting and testing sufficient reference subjects per partition and should also include inter-ocular symmetry. Subjects should be matched to the patient population in demographic factors. If reference data from elsewhere are used, for example, manufacturers’ data or published data, they must be verified as appropriate for local use with an understanding of possible limitations and how the reference limits were defined. Reference limits should be constructed using non-parametric or robust techniques to enclose the central 95% of values, i.e., the 2.5th and 97.5th percentile. Inter-ocular comparisons are particular sensitive if there is unilateral or asymmetrical pathology. The VEPs in adults are relatively stable but age-specific reference limits are essential for children and the elderly population. In longitudinal studies, subject-specific reference values may be more useful than a comparison with a reference group (cross-sectional population-based control values) to decide whether a response has changed by a clinically meaningful amount. Such assessments should consider the consistency of stimuli and recording methods, and inter-session variability due to other factors e.g., age or pupil diameter etc.

## Optional ISCEV standard methods

### Simultaneous PERG and pattern-reversal VEP

Since most of the pattern-reversal VEP signal is mediated through the macular fibers of the optic nerve, functional assessment of central retinal function is crucial for the reliable interpretation of abnormal pattern-reversal VEP. An ISCEV Standard multifocal ERG may provide an indication of macular cone function [3]. Alternatively, macular and retinal ganglion cell function may be conveniently assessed by either additional recording of the PERG or simultaneously recording the PERG and pattern-reversal VEP, described below and also in the ISCEV Standard for clinical pattern ERG [5].

For simultaneous recordings a check width of 0.8–1.0° (48’-60’) and a reversal rate of 3 rps is specified. This check width is within the range for both the ISCEV Standard PERG (0.8° ± 0.2°) and the larger check width for ISCEV Standard pattern-reversal VEP (1° ± 0.2°). The mean of the width and the height of the stimulus field should be at least 15°. A reversal rate of 3 rps is at the lower limit for the ISCEV Standard PERG (4 rps ± 1.0 rps) and is sufficiently close to the ISCEV standard pattern-reversal VEP (2 rps ± 0.2 rps), to enable transient pattern VEP recordings. The acquisition time window of 250–285 ms allows that both the PERG and pattern-reversal VEP can be recorded simultaneously on separate channels. Monocular simultaneous PERG and pattern-reversal VEP acquisition may be helpful in cases where patient compliance is limited or when longer test sessions with multiple tests may not be possible, or when active defocus suspected or to align fixation in cases of strabismus. Reports should explicitly state the use of simultaneous recording, the stimulus check width and reversal rate, and interpretation based on reference data to identical stimuli.

## Non-standard methods

### Multi-channel VEP recording

Multi-channel VEP recording is necessary to assess chiasmal and post-chiasmal visual pathway dysfunction and to detect optic nerve misrouting at the chiasm. An asymmetrical trans-occipital distribution of the monocular VEP over the multi-channel array of electrodes, for example lower amplitude and/or longer peak time over one occipital hemisphere, can localize dysfunction. If the VEP abnormality (inter-hemispheric asymmetry) is over the same hemisphere irrespective of which eye is stimulated, it is called an uncrossed asymmetry consistent with retro-chiasmal dysfunction. If the VEP abnormality changes to the other hemisphere when the other eye is stimulated, it is called a crossed asymmetry, in keeping with chiasmal dysfunction or visual pathway misrouting at the chiasm.

#### Electrode placement

A minimum of three active electrodes are needed to detect lateral occipital asymmetries; a midline active electrode at Oz and two lateral electrodes placed at O_1_ and O_2_ are recommended. All three active electrodes may be referenced to Fz. Additional electrodes placed at PO_7_ and PO_8_, also referred to Fz may increase sensitivity to lateral trans-occipital distribution asymmetries. The positions of the trans-occipital array of electrodes are illustrated in Fig. 1B.

#### Stimulus specifications

Both pattern and flash Standard stimuli can be used to record multi-channel VEPs. For greater specificity pattern-reversal stimulation can be confined to one hemisphere by presenting pattern monocularly in the temporal and nasal central half-field. For half-field stimulation, the stimulus covers only half of the screen, while the other half of the screen is blank. Care must be taken that the fixation point is centered in the middle, on the border between the stimulated and blank part of the screen. Alternatively, it is possible to use the method in which the entire field of the screen is stimulated, and the fixation is moved to the right or left edge of the screen (when the left edge of the screen is fixed, the right half-field is stimulated and vice versa).

#### Paradoxical lateralization

Caution is needed when interpreting multi-channel pattern-reversal VEPs because of paradoxical lateralization, in which the response recorded at a lateral scalp location is generated by activity in the contralateral hemisphere of the brain. This phenomenon occurs with a large field, large (1°) check width pattern-reversal stimulus and common reference recording to Fz. Paradoxical lateralization is important for interpretation of half-field pattern-reversal testing. The pattern-reversal VEP N75, P100 and N145 components to right and left half-field stimulation are seen over the right and left hemisphere respectively i.e., opposite to the occipital hemisphere being stimulated.

#### Reference ranges for multi-channel VEPs

For all stimulus conditions, reference data should include amplitude and peak time comparisons for each eye over left and right occipital channels—the VEP would be expected to be the same for each eye over the same hemisphere. It is also necessary to describe any abnormal waveshape of the VEP, especially the reversal of polarity on one of the lateral channels, compared to the other. When using half-field testing, reference data should also include a comparison of relative interocular amplitude and peak time differences from either half-field, as well as monocular half-field amplitude and peak time difference from either eye.

#### Other technical considerations

The difference over the lateral channels can be emphasized by introducing a differential channel (the signal from the right lateral channel is subtracted from the left channel, or vice versa). In this way, a defect in the chiasmal region is easier to identify, since the signal of this differential channel is of exactly the opposite polarity when compared between the eyes. This is especially useful for the detection of chiasmal misrouting with flash and pattern onset/offset stimulation.

### Pediatric VEP recording

In principle, the stimulation and recording methods specified by this Standard can be applied to all populations. However, in infants, young children and in individuals of all ages with compliance difficulties, modifications to Standard VEP recording methods and testing strategies may be required to optimize the quality of the result and the pertinence to diagnosis and to visual assessment.

#### Electrode placement

The amplitude of the pattern-reversal VEP P100 to large checks is larger over the inion (Iz) than Oz in children up to 8 years, and use of an additional lower midline electrode at the inion (Iz) can be informative. Some infants have atypical occipital head shapes or positional plagiocephaly and routine trans-occipital electrode arrays can reassure that the optimal VEP is being recorded, as well as assessing chiasmal and post-chiasmal pathway function (ref. 6.1).

#### Check widths and field size

A range of check widths larger than the ISCEV standard large check (1°) may be needed to produce consistent pattern VEPs in babies and infants under 4 months as spatial sensitivity is still maturing. Smaller check widths help monitor visual development after 9 months. A larger pattern stimulus field (such as 30°) can maximize a child’s fixation time for capturing a pattern VEP and may also help with interpretation of trans-occipital recordings.

#### VEP waveshape in childhood

VEP waveform shape to all stimuli changes with age. The pattern-reversal VEP to both large and small check widths is dominated by a single positive peak, but the P100 to the large check width is detected at a younger age than P100 to small check width. Pattern onset/offset VEPs in infancy and childhood to the large check width typically comprise a single positive peak which can dominate the onset VEP waveform until 20 years of age. With increasing age, the negative C2 becomes more prominent, but the depicted C1, C2, C3 components of the ISCEV Standard onset VEP waveform to Standard check widths (Fig. 2B) may not appear until the age of 40 years. An onset VEP to small check widths is distinguished by a more prominent negative C2 at earlier ages and earlier C1 and C2 peak times than to large check widths. Flash VEP waveforms can be polyphasic and in children it may not be possible to identify P1 and P2. Describing the main or largest positive peak usually enables a meaningful inter-ocular comparison.

#### Compare gestational age to reference range

VEPs in infants and children should be compared with appropriate age-related reference values after correction for prematurity i.e., compare reference values for the gestational age of a young infant. In typically developing children with good vision the pattern-reversal VEP P100 peak time drops rapidly from birth to stabilize within 10% adult values by 27 and by 34 weeks of gestational age for large (1°) and small check widths (0.25°), respectively.

#### Sweep duration and stimulation rate for infants

Because of longer VEP peak times in infancy (~ 200 ms in first 1 month of life) it is helpful to record the VEP of young infants with a sufficient sweep duration to record the full VEP waveform; for example, for infants less than 8 weeks 0.5 Hz stimulation rate and sweep duration of 400 ms will prevent stimulus response overlap.

#### Other technical considerations

Pediatric VEPs should be recorded when the infant or child is alert and attentive. Sedation and anesthesia can change the shape, amplitude and peak time of the VEP. Direct interaction with the child can help maintain attention and fixation, and two testers are beneficial: one to work with the child and the other to control data acquisition. Data quality and reliability will be improved if recording can be paused or interrupted when fixation wanders and then resumed as the child resumes adequate fixation. An unobtrusive camera mounted close to the TV to monitor fixation can support interrupted recording. To facilitate compliance, an infant may view the stimulus while held on a lap or over a shoulder. The room lights can be extinguished to remove extraneous distractions. The order of stimulus presentation should be flexible and selected to ensure that VEPs most critical to the diagnostic question are obtained within an individual child’s attention span. Binocular pattern stimulation facilitates attention and fixation, and may be useful to evaluate overall visual function, but monocular testing to at least one stimulus is desirable to assess the visual pathway function of each eye.

It is particularly important to replicate VEPs in children to ensure that the response measured is a reliable signal and not an artifact. The inclusion of non-stimulus trials is recommended for easier differentiation of real signal from the noise. Reports should note the degree of cooperation and arousal of the child, as well as any intrusion of EEG, which could mimic, augment or confound VEPs. When pattern VEPs cannot be recorded reliably, or are not measurable, a technically adequate flash VEP, which is less dependent upon fixation, can usually be achieved. During monocular flash VEP stimulation in young children a simultaneous recording of flash skin ERGs from both eyes can be used to detect stray light stimulating the occluded eye and can inform interpretation in the stimulated eye. After recording monocular flash VEPs in a drowsy infant it can be beneficial to repeat testing of the first eye immediately after the second to capture a similar state of arousal.

As for adults, multi-channel occipital recording channels are important for the diagnosis of chiasmal and post-chiasmal dysfunction. Children often present with a clinical question, such as unexplained poor vision, that does not indicate chiasmal or hemisphere pathology specifically. For such children it is helpful to record monocular VEPs from a trans-occipital array of electrodes to provide complementary information, to detect and importantly exclude pathology, irrespective of the stimulus modality used. The sensitivity of a VEP to detect some chiasmal conditions, such as the misrouting of albinism, changes with stimulus and age, being highest with flash in infancy to onset/offset VEPs in later childhood [7], but between these ages will vary for individual children.
